# Genomic Analysis of Lumpy Skin Disease Virus from Western and Central Africa Suggests a Distinct Sub-Lineage Within the 1.2 LSDV Cluster

**DOI:** 10.3390/pathogens14090922

**Published:** 2025-09-12

**Authors:** John Fadele, Olusola Ogunsanya, Oluwatobi Adedokun, Akeemat Ayinla, Mbitkebeyo Pami, Ayotunde Sijuwola, Femi Saibu, Harouna Soumare, Urbain Fanou, Corrie Brown, Bonto Faburay, Christian Happi, Anise Happi

**Affiliations:** 1 Institute of Genomics and Global Health, Redeemer’s University, Ede 232102, Osun State, Nigeria; fadele15895@run.edu.ng (J.F.); ogunsanyao@run.edu.ng (O.O.); adedokunolu@run.edu.ng (O.A.); hakimattayinla@gmail.com (A.A.); mbitkebeyo17982@run.edu.ng (M.P.); sijuwolaa@run.edu.ng (A.S.); saibum@run.edu.ng (F.S.); soumareh@run.edu.ng (H.S.); happic@run.edu.ng (C.H.); 2 Department of Biological Sciences, Redeemer’s University, Ede 232102, Osun State, Nigeria; 3 Clinique Veterinaire Bon Pasteur, Fidjirosse, Cotonou 00229, Benin; urbainfanou@yahoo.fr; 4 LifeStock International, Athens, GA 30606, USA; corrie@lifestock.org; 5 Foreign Animal Disease Diagnostic Laboratory, U.S. Department of Agriculture, Animal and Plant Health Inspection Service, National Bio and Agro-Defense Facility, USDA, Manhattan, KS 66502, USA; bonto.faburay@usda.gov

**Keywords:** Lumpy Skin Disease Virus (LSDV), genomic analysis, phylogenetic analysis, SNP mutations, vaccine efficacy, vector transmission, Africa, poxviruses, ankyrin repeat proteins, Kelch-like proteins

## Abstract

Lumpy Skin Disease Virus (LSDV) is a transboundary pathogen that affects cattle, causing significant economic losses, particularly in Africa and Asia. While the virus was originally endemic to sub-Saharan Africa, it has rapidly spread to Europe, the Middle East, and Asia, necessitating comprehensive genomic surveillance. Despite LSDV’s African origins, genomic data from West and Central Africa remain scarce, limiting insights into regional viral evolution and vaccine compatibility. In this study, molecular detection of LSDV was carried out on cattle samples from Nigeria, Cameroon, and Benin. However, comparative genomic analysis was performed using two near-complete LSDV genomes obtained from Cameroon. Phylogenetic evaluation revealed that LSDV strains from Nigeria and Cameroon cluster within the classical 1.2 lineage. Furthermore, the two sequences from this study cluster with the only publicly available sequence from West and Central Africa, supporting earlier findings of the presence of a West/Central African sub-lineage. Functional genomic analysis identified mutations in genes encoding ankyrin repeat Kelch-like proteins, and envelope proteins involved in immune evasion and viral virulence, raising concerns about vaccine effectiveness. Furthermore, the detection of LSDV in flesh flies (*Sarcophaga* spp.) underlines their potential role in virus transmission. These findings highlight the importance of genomic monitoring and targeted surveillance.

## 1. Introduction

Lumpy Skin Disease Virus (LSDV) is a transboundary pathogen affecting cattle and causing significant economic losses, particularly in Africa and Asia. The disease leads to decreased milk production, hide damage, reproductive failures, and high treatment and vaccination costs [1,2]. In regions where livestock forms a central component of rural livelihoods and food security, such as sub-Saharan Africa, these impacts are especially devastating [3]. The global economic burden of LSDV has intensified with its expansion beyond Africa, with outbreaks in Asia alone estimated to have caused losses of approximately USD 1.45 billion [4]. In Ethiopia, the median economic loss was estimated at USD 375 per dead animal (ranging from USD 325 in local Zebu to USD 1250 in Holstein-Friesian crossbred cattle), while the median total herd-level loss from an outbreak was USD 1176, varying from USD 489 in subsistence farms to USD 2735 in commercial farms [5], while the 2022 outbreak in Pakistan affected over 5 million farmers [5].

Initially endemic to sub-Saharan Africa, LSDV has now been reported across the Middle East, Asia, and Europe, with a rapid geographic expansion observed since 2012, when more than 98% of outbreaks were recorded [2]. Recent outbreaks in countries such as Russia [6], China [7], Turkey [8], and Nigeria [9] highlight its widespread distribution. Environmental factors such as temperature, precipitation, vector abundance, and land use influence its transmission patterns [10]. Additionally, informal livestock trade and the emergence of recombinant strains have been implicated in recent outbreaks, although the extent to which the properties of the recombinant strain contribute to dispersal or sustained spread remains uncertain [11].

LSDV is primarily vector-transmitted, with mosquitoes [12,13], stable flies [14,15], and ticks [16,17] identified as key mechanical vectors. While direct transmission between cattle is considered rare, seasonal outbreaks often coincide with peak vector activity [2]. There is also emerging evidence for venereal, airborne, and alimentary transmission [18]. Clinically, LSD presents with fever, nodular skin lesions, emaciation, and reproductive failure, leading to substantial morbidity [19].

Vaccination remains the most effective control strategy against LSDV, although vaccine efficacy may vary by strain and production setting. In Ethiopia, the KS-1 vaccine (an attenuated LSDV strain previously thought to be SPPV) has shown incomplete protection, with outbreaks reported in vaccinated dairy herds [20]. By contrast, the Neethling vaccine strain, which has been widely used in Israel and parts of Europe, demonstrated stronger protection, with an average efficacy of 79.8% during the 2016–2017 Balkan epidemic [21,22]. However, the emergence of new LSDV variants due to genetic mutations and recombination, especially between vaccine and field strains, has raised concerns about current vaccine efficacy [23,24,25].

LSDV has a large double-stranded DNA genome of approximately 151 kilobase pairs, encoding about 156 genes involved in replication, immune evasion, and host range [26]. Although genetically conserved, LSDV displays sufficient diversity through mutations and recombination to result in distinct viral lineages with different epidemiological profiles [27]. Recombinant strains have demonstrated altered transmission routes and enhanced oronasal spread, complicating control efforts. Studies in Uganda and Nigeria have shown genetic similarities between African and Eurasian strains [28], and genomic analysis of LSDV isolates from India and Bangladesh revealed close similarity to historical Kenyan strains, suggesting a common exotic source [29], emphasizing the need for continuous genomic monitoring.

Despite the African origin of LSDV and its continued burden in African cattle populations, there is a notable shortage of complete genome sequences from West and Central Africa. To date, only one full genome has been reported from this region, limiting our understanding of local viral diversity and hindering efforts to assess vaccine compatibility and strain evolution. Given the increased frequency and severity of outbreaks, it is essential to fill this knowledge gap through regional genomic surveillance and characterization.

This study aims to address this gap by characterising LSDV strains circulating in Nigeria, Cameroon, and Benin. Through molecular detection, sequencing, and comparative genomic analysis, we provide insights into the evolutionary dynamics of LSDV in West and Central Africa and their implications for disease control and vaccine development.

## 2. Materials and Methods

### 2.1. Animal Sample Collection

**Figure 1:**
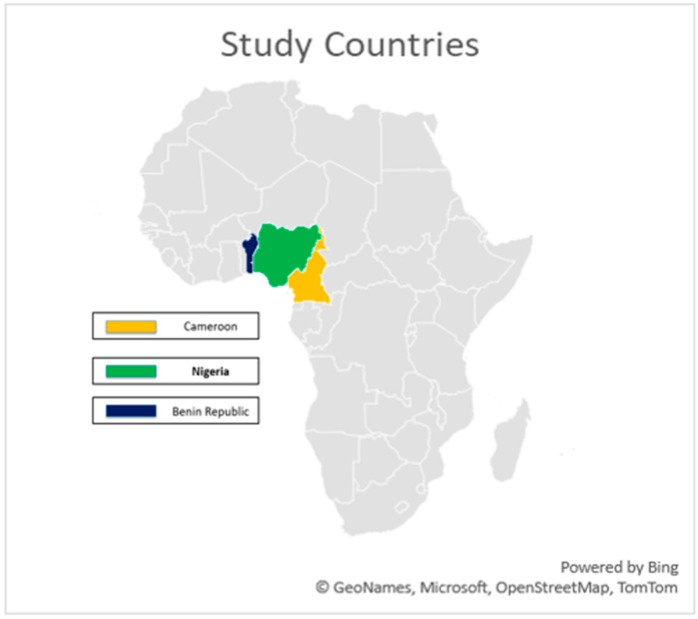
Map of Africa highlighting the study area.

Samples were collected from cattle from three countries (Nigeria, Cameroon and Benin). Nigeria shares boundaries with both Cameroon and Benin (Figure 1). The sampled animals were from farms, cattle markets and abattoirs. These locations were areas where contact or local veterinarians reported cases of cattle with pox-like lesions. The sampling activity was done by experienced veterinarians who purposefully sampled animals with skin nodules of varying sizes, severity and stages, typical of LSD. Samples collected include skin scabs, nodule aspirates, and oral and nasal swabs (the oral and nasal swabs were pooled into one sample for each animal). Collected samples were stored in DNA/RNA shields and transferred into mobile freezers (−4 °C). Then, they were transported to the Institute for Genomics and Global Health (IGH), Redeemer’s University, Ede, Nigeria, and stored at −80 °C until laboratory analysis.

### 2.2. Fly Collection and Processing

Flies were also collected from sampled sites. The flies were captured using fine-mesh scoop nets and baited conical traps containing FLY IN BAIT^®^ and decomposing organic matter. Captured flies were immediately transferred into labelled zip lock bags and transported in an ice box to maintain the cold chain. Upon arrival at the laboratory, samples were stored at −20 °C. Identification was carried out based on morphological characteristics, including sex differentiation and species classification using key features of the head, thorax, abdomen, and general body structure. Processed flies were grouped into pools, each consisting of 10 flies of the same sex and species (fly type). All pools were stored in 1.5 mL safe-lock Eppendorf^®^ tubes containing 500 µL of RNA/DNA Shield for downstream molecular analysis.

### 2.3. DNA Extraction and qPCR

The extraction process utilised the QIAamp DNA Mini kit from Qiagen (Hilden, Germany) in accordance with the manufacturer’s instructions. The methodologies varied depending on the sample type, particularly involving the homogenisation of skin scabs. A TaqMan-based RT-qPCR analysis was conducted using specific primers (LSDV-F: TGAATTAGTGTTGTTTCTTC; LSDV-R: GGGAATCCTCAAGATAGTTCG) and a probe (LSDV-P: FAM-TGCCGCAAAATGTCGA-MGB) to target the P32 gene of the LSD virus. RT-qPCR amplifications were performed in a 25 µL reaction mix containing 5 µL of extracted sample nucleic acid or template controls, along with 20 µL of the prepared master mix. The PCR master mix comprised 6.25 µL of TaqPath 1-Step RT-qPCR reaction mix, 1 µL of each primer, 0.5 µL of probe, and 11.25 µL of nuclease-free water. The thermocycling conditions for the PCR were set at 50 °C for 2 min, followed by 95 °C for 5 min, and then 40 amplification cycles (95 °C for 15 s, 58 °C for 15 s). The reaction was performed on a Lightcycler 96 by Roche Sequencing, and a CT value < 40 was considered a positive sample.

### 2.4. Library Construction, Hybridisation Capture and Sequencing

We performed library construction following the methods used by Kapoor et al. [30]. Libraries were constructed using the Twist library preparation kits (Twist Biosciences, South San Francisco, CA, USA). The indexed libraries were pooled and then set up for liquid hybridisation capture using the VirCapSeq-VERT probe by employing the Twist Fast Hybridisation Reagents (Twist Biosciences, USA). The enriched and purified pools were quantified and thereafter normalised. Paired-end sequencing was carried out using a P3 (300-cycle) cartridge and flowcell on the Illumina NextSeq 2000 instruments (Illumina, San Diego, CA, USA).

Sequencing was performed on 28 samples, which included one representative sample from each of the 14 animals testing positive by qPCR, the single fly pool positive for LSDV, 10 skin scab samples from animals either in direct contact (i.e., sharing the same farm) with confirmed cases, two skin scabs from cattle showing extreme and generalized skin lesions that field veterinarians clinically diagnosed as LSD, and a pool of ticks collected from one qPCR-positive animal. In instances where more than one sample type tested positive from the same animal, the sample with the lowest Ct value was chosen.

### 2.5. Statistical Analysis

Statistical analysis to determine the level of significance of the rate of positivity across sampled countries and the sample types was carried out using IBM SPSS 27. *p*-value < 0.05 was considered significant.

### 2.6. Bioinformatics Analysis

The FastQ files generated from sequencing were first taken through FastQC to check for the quality of the reads. Afterwards, the reads were trimmed using Trimmomatic to remove poor-quality reads and adapters. This was followed by taxonomic classification using Kraken2 [31]. The pre-processed reads were aligned to the reference genome (NC003027) using BWA (Burrows-Wheeler Aligner) [32]. Finally, a consensus genome was generated using iVar [33]. This involved aligning the BAM files, variant calling, and constructing a high-confidence consensus sequence, where bases were only called at positions with a minimum read depth of 10× and a base frequency of at least 50%. Positions not meeting these criteria were masked with ‘N’.

Phylogenetic analysis was done using a final dataset of 34 sequences, consisting of the two study sequences from Cameroon and 32 sequences retrieved from the NCBI virus database. These 32 sequences were selected as representatives from the different LSDV clusters as outlined by [34]. The multiple sequence alignment was performed using the online MAFFT tool (MAFFT alignment and NJ/UPGMA) [35]. The maximum likelihood tree was constructed using IQTREE2 [36], and iTOL (Interactive Tree of Life) was used for visualisation and annotation [37].

Variant analysis was performed to identify single-nucleotide polymorphisms (SNPs) and insertions or deletions (Indels) using NUCmer version 3.1, a component of the MUMmer package [38]. Each of the two newly assembled Cameroonian genomes (CAYDCAT29 and CAYDCAT30) and four representative genomes from sub-lineages within the LSDV cluster 1.2 (Dagestan2015, Warmbaths2000, Nigeria2018, and Israel2012) [34] were individually aligned to the LSDV reference genome (NC003027). SNPs and Indels were identified for each genome relative to the reference, and the resulting variant profiles from all six genomes were subsequently compared to determine similarities and differences across the 6 genomes. To refine the alignment data from NUCmer, Delta-filter (a built-in post-processing tool in MUMmer) was used to keep high-quality matches and to eliminate weak alignments. Additionally, AWK (a text-processing tool) was used to remove ambiguous SNPs, ensuring high-quality variant calls [39].

The two sequences from this study were annotated, and the functional effects of mutations were predicted using SnpEff [40]. For this analysis, a custom SnpEff database was created using the reference genome together with its corresponding GFF file, which contains the coordinates of the 156 putative LSDV genes as well as the translated amino acid sequences. SnpEff compares each query genome to the reference through pairwise alignment, determines how each gene aligns with its corresponding reference gene, and then identifies sequence variations. Based on the gene and codon information in the GFF file, the software predicts the potential functional impact of each detected mutation. The result generated from NUCmer and SnpEff was converted to CSV files, and visualisations were done using R.

## 3. Results

### 3.1. Sample Distribution

A total of 172 cattle were sampled across the three countries. In Cameroon, 57 cattle were sampled from eight farms and one cattle market. In Benin, 55 cattle were sampled from nine farms. In Nigeria, 60 cattle were sampled from two abattoirs. Overall, swabs constituted the largest proportion of sample types collected (53.5%), followed by skin scabs (43.1%) and nodule aspirates (3.4%). In Benin and Nigeria, no nodule aspirates were collected. In Cameroon, nodule aspirates were collected from 10 (10.6%) animals (Table 1). The distribution of pooled fly samples varied across the three countries (Table 2). Five fly pools each from Nigeria and Cameroon were analysed, while 31 pools of flies from Benin were analysed.

### 3.2. PCR Results

An animal was confirmed positive when at least one of its collected samples tested positive by qPCR. In total, 14 of the 172 animals tested (8.1%) were positive. Of these, five animals yielded more than one positive sample type. Seven animals tested positive for skin scab alone, one for nodule aspirate alone, and one for oral/nasal swab alone. Full qPCR results, including Ct values for positive samples, are provided in Supplementary Materials S1 (qPCR result). Positive animals were from three farms (3/8) and one cattle market (1/1) in Cameroon, one farm (1/9) in Benin, and one abattoir (1/2) in Nigeria. Among the countries studied, Cameroon had the highest positivity rate, with 12 out of 57 animals testing positive (21.1%). In contrast, Nigeria and Benin showed significantly (χ2 = 19.01, *p* < 0.001) lower LSDV PCR positivity rates of 0.8% (1/120) and 1.2% (1/83), respectively, compared to Cameroon (Table 3). PCR results also varied by sample type. Skin scabs showed the highest positivity rate of 9.4% (12/128) among the sample types (Table 3). However, the association between sample type and PCR positivity was not statistically significant (χ2 = 3.9, *p* = 0.139). Of the 41 fly pools tested by PCR, only one fly pool (flesh fly pool from Cameroon) tested positive for LSDV by PCR (Table 2).

### 3.3. Sequencing and Phylogenetic Analysis

Of the 28 samples subjected to sequencing, 14 yielded reads mapping to LSDV (Supplement S2). Notably, one sample with LSDV reads was PCR-negative, although the read count was substantially low, whereas three PCR-positive samples did not yield any LSDV reads through sequencing. To ensure reliability, a cut-off of >500 read count for LSDV was applied, below which samples were excluded from downstream assembly. Eight samples met this threshold (Supplement S3). Among these, genome coverage ranged between 1.17% and 98.06% of the LSDV reference genome (NC003027), with mean read depths varying from 1.08× to 123.27×.

**Figure 2:**
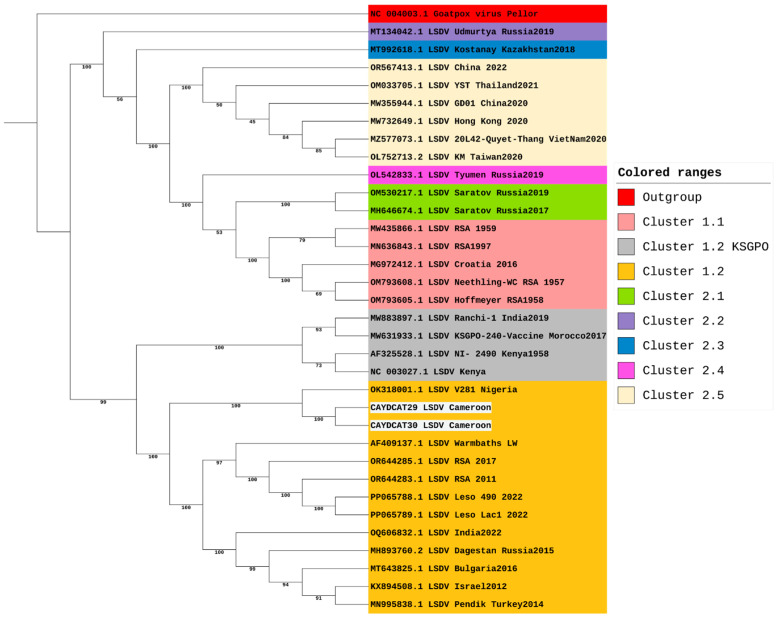
Phylogenetic Analysis of LSDV Genomes including study samples from Cameroon.

Two samples (CAYDCAT29 and CAYDCAT30) achieved near-complete genomes, covering 91.53% and 98.06% of the reference genome with average read depths of 93.99× and 123.27×, respectively. Notably, both near-complete genomes originated from Cameroon, whereas the samples from Nigeria and Benin produced either very low or no LSDV-aligned reads. The maximum likelihood tree (Figure 2) was constructed using 34 sequences, including the two LSDV full genomes generated during this study from Cameroon and 32 from the NCBI virus database. LSDV strains are sorted into 7 clusters, with clusters 1.1 and 1.2 being the classical strains and clusters 2.1 to 2.5 containing vaccine recombinant strains. Phylogenetic analysis shows that the two Cameroon genomes cluster together within cluster 1.2 and are closely related to other African strains but are distantly related to classical strains in cluster 1.1 and vaccine recombinant strains. These two sequences form a clade with the only LSDV strain (whole genome) from Nigeria available on NCBI (OK318001). This shows that the Nigerian strain is the most closely related to the two sequences from this study.

**Figure 3:**
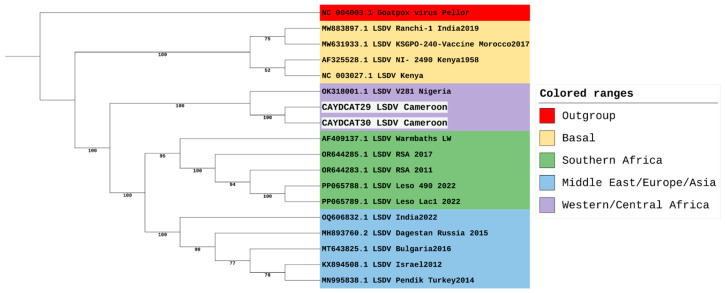
Phylogenetic Analysis of LSDV Cluster 1.2, including study samples from Cameroon.

Being the most divergent of the 7 LSDV clusters, a maximum likelihood tree focusing on cluster 1.2 was constructed to focus on its three established sub-lineages (Figure 3). However, our sequences from Cameroon, together with the sequence from Nigeria, do not all fit into any of the three sub-lineages of cluster 1.2. This suggests that while the LSDV strains from Nigeria and Cameroon might be closely related to the strains from Southern Africa, and some strains from Europe, Asia, and the Middle East, these three strains appear slightly distinct from other strains within cluster 1.2. Hence, we suggest that these three strains could belong to a separate sub-lineage and could be tagged the Western/Central African sub-lineage within the LSDV 1.2 cluster.

### 3.4. Comparative Genomic Analysis of Cameroonian LSDV Genomes Reveals SNP and Indel Variations

Quantification of the single-base substitutions (SNPs), insertions, and deletions in each sequence (Table 4) revealed that the two newly assembled Cameroonian LSDV genomes displayed fewer mutations compared to the other 4 reference representatives. CAYDCAT29 showed 124 single-base substitutions, 14 insertions, and 22 deletions, while CAYDCAT30 showed a higher number of substitutions (*n* = 136), with 24 insertions and 25 deletions. Among the genome representatives, Dagestan2015 showed the highest number of single-base substitutions (*n* = 147), suggesting a more divergent lineage within cluster 1.2. Warmbaths2000 showed the highest number of insertions (*n* = 78), while Israel2012 presented the highest number of deletions (*n* = 45). These differences reflect distinct evolutionary trajectories within the cluster.

**Figure 4:**
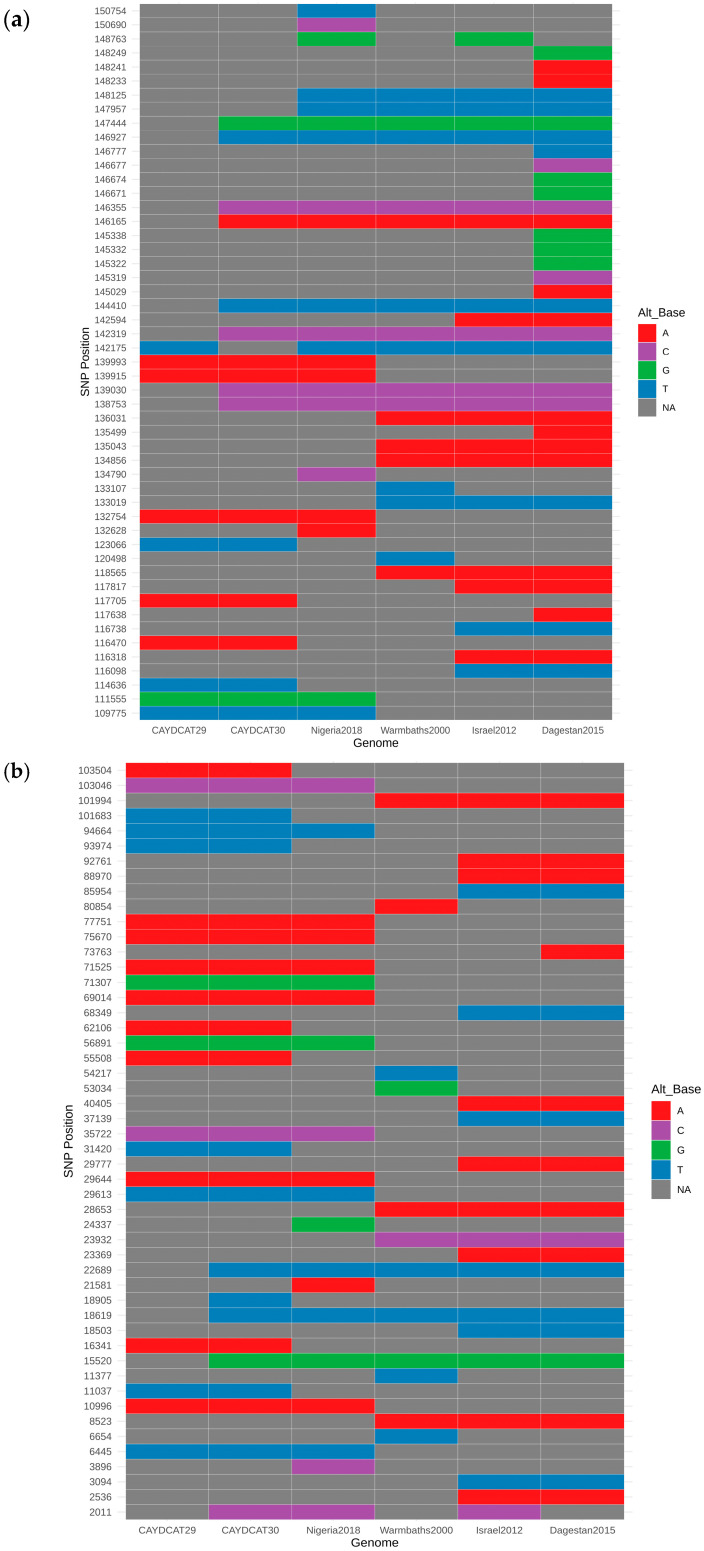
(**a**,**b**) SNP heatmaps showing 101 genomic positions with variation across the six compared LSDV genomes. Each column represents an individual genome, and each row represents a SNP locus (each nucleotide is represented by a specific colour, and NA is used when an SNP is absent). CAYDCAT29 and CAYDCAT30 exhibited nearly identical SNP patterns, with a high degree of overlap with the Nigeria 2018 strain.

A total of 101 unique SNP positions were identified across the six genomes, spanning both coding and non-coding regions. SNP heatmap plots (Figure 4a,b) revealed a high degree of conservation between the Cameroonian genomes. CAYDCAT29 and CAYDCAT30 exhibited nearly identical SNP signatures, further supporting their close relationship. Notably, several SNPs were conserved between the Cameroonian LSDV genomes and Nigeria2018, suggesting a shared sub-lineage or recent common ancestor.

**Figure 5:**
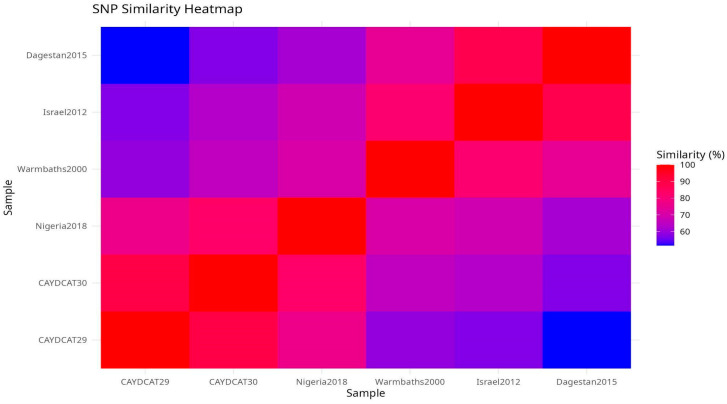
Pairwise SNP similarity heatmap scores based on the Jaccard index, calculated from shared and unique SNPs across all genome pairs. CAYDCAT29 and CAYDCAT30 exhibit the highest similarity (0.898), followed by Nigeria2018. Lower similarity values with Warmbaths2000, Israel2012, and Dagestan2015 reflect divergent sub-lineages.

To explore overall genomic similarity, we constructed a heatmap (Figure 5) using the Jaccard similarity coefficient, which quantifies the proportion of shared SNPs relative to the total set of unique SNPs between genome pairs. This approach captures both convergent and divergent SNP signatures across the genomes. CAYDCAT29 and CAYDCAT30 exhibited the highest similarity (>85%), consistent with a shared origin. Both genomes also have close SNP patterns with Nigeria2018 (Jaccard similarity between 75 and 85%), further supporting their placement within a Central/West African subset of LSDV cluster 1.2. In contrast, Warmbaths2000, Israel2012, and especially Dagestan2015 showed lower similarity to the Cameroonian LSDV genomes (Jaccard indices < 62%), suggesting more distant evolutionary relationships. These patterns confirm that the Cameroonian strains are genetically distinct but most closely related to Nigeria2018, supporting the hypothesis of regional continuity in virus evolution within cluster 1.2.

### 3.5. Functional Impact of Genetic Variation in Cameroonian LSDV Genomes

**Figure 6:**
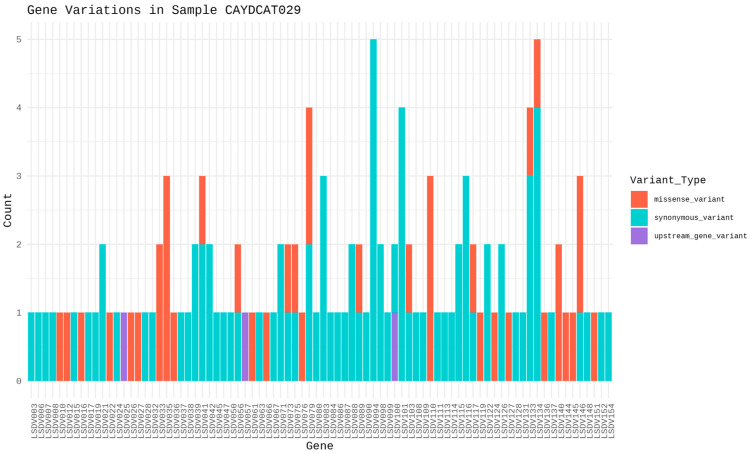
Gene variation profile in sample CAYDCAT29. Distribution of gene variants across all genes, showing the count of missense variants, stop gained, synonymous variants, and upstream gene variants.

**Figure 7:**
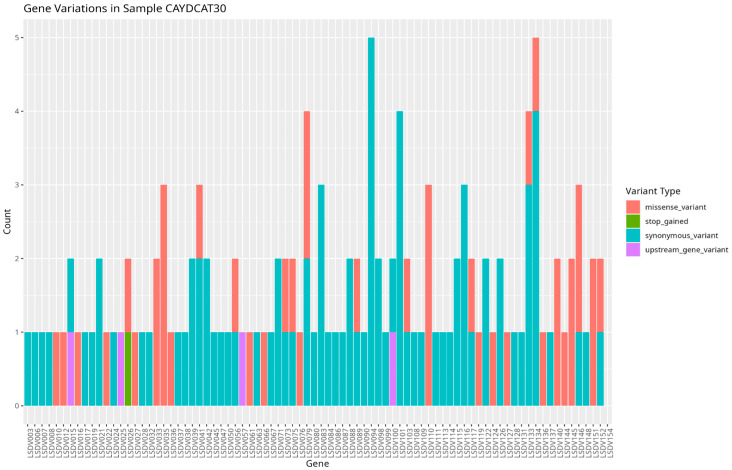
Gene variation profile in sample CAYDCAT30. Distribution of gene variants across all genes, showing the count of missense variants, stop gained, synonymous variants, and upstream gene variants.

To assess the functional characteristics of the genetic variations in the Cameroonian LSDV genomes, we examined the distribution of mutations across affected genes and their potential functional consequences. A total of 82 genes in CAYDCAT29 (Figure 6) and 81 genes in CAYDCAT30 (Figure 7) were found to contain at least one sequence variant (Table 5). Among these, 23 genes in CAYDCAT29 and 25 genes in CAYDCAT30 harboured two or more mutations. Furthermore, 35 genes in CAYDCAT29 and 37 genes in CAYDCAT30 encoded non-synonymous mutations, and only one gene, LSDV154, was uniquely mutated in CAYDCAT29, while no genes were uniquely affected in CAYDCAT30. Overall, 126 and 133 mutations were functionally annotated in CAYDCAT29 and CAYDCAT30, respectively. The most prevalent class of coding variation was synonymous substitutions (*n* = 86 in CAYDCAT29; *n* = 85 in CAYDCAT30), followed by missense mutations (*n* = 40 and 43, respectively). Notably, a single stop-gain mutation was observed in LSDV026 of CAYDCAT30 but was absent in CAYDCAT29. Additionally, both genomes carried a small number of upstream gene variants (*n* = 3 in CAYDCAT29; *n* = 4 in CAYDCAT30). The full list of annotated mutations is provided in Supplements S4 and S5 for CAYDCAT29 and CAYDCAT30, respectively.

Furthermore, our study identified several genes harbouring missense variants that may influence viral gene expression and immune suppression. Missense variants were observed in genes encoding ankyrin repeat proteins LSDV012 and LSDV145, which have been reported to inhibit interferon-induced proteins, particularly IFIT1, thereby enhancing viral replication [41]. Similarly, mutations were identified in LSDV144 and LSDV151, which encode Kelch-like proteins involved in protein–protein interactions that facilitate immune evasion [42]. Missense variants were also detected in LSDV140, homologous to poxvirus N1R/p28, which is known to suppress apoptosis and promote viral virulence [43]. Additionally, the phospholipase-D-like protein LSDV146 plays a role in viral dissemination [44,45], also exhibited missense variation.

Among structural proteins, our study identified missense variants in the gene encoding the putative extracellular enveloped virus (EEV) maturation protein LSDV027, which is essential for viral dissemination as inferred from UniProt. Similarly, missense variants were found in the virion core protein genes LSDV041 and LSDV103, which undergo proteolytic processing during the transition from immature virion to mature virion [26,42,46]. Missense variants were also present in LSDV073 and LSDV075, which encode putative viral membrane proteins with putative roles involving viral entry, membrane fusion, and virion assembly [42].

Several genes encoding viral enzymes also had mutations (missense variants). These mutations can potentially affect viral transcription and replication. Variants were observed in LSDV036 and LSDV119, which encode RNA polymerase subunits and are involved in viral mRNA synthesis [26]. Missense variations were also present in LSDV079 and LSDV089, which encode mRNA-capping enzyme subunits responsible for ensuring mRNA stability and efficient translation [47]. Additionally, missense variants were detected in LSDV066, a thymidine kinase essential for nucleotide metabolism and viral replication [48], and LSDV133, a DNA ligase-like protein that plays a role in genome integrity by repairing and ligating DNA strands [49].

Our study also identified upstream gene variants in several essential viral genes. Variants were observed in LSDV015, which encodes an IL-18 binding protein known to modulate immune responses by inhibiting IL-18 activity [50]. Upstream variants were also present in LSDV025, which encodes a serine/threonine kinase involved in viral replication [51]. Additionally, mutations were found in LSDV057 and LSDV100, which encode a putative virion core protein and an intracellular mature virus membrane protein, respectively, potentially influencing virion assembly and infectivity [52,53].

## 4. Discussion

This study detected LSDV in cattle from Benin, Cameroon, and Nigeria. Despite low percentage positivity, we added two new LSDV genomes in the global database and identified a potentially new West and Central Africa sub-lineage of the virus. Only the Nigerian strain of LSDV in the GenBank is closely related to the sequences from this study.

Consistent with recent findings [54], our genomic analysis demonstrates that the Cameroonian sequences cluster with the Nigerian 2018 isolate (OK318001), together forming part of the newly defined West/Central African sub-lineage of LSDV (clade 1.2.3). This supports the existence of a distinct subgroup that has circulated in the region for decades. Importantly, given the scarcity of genomic data from West and Central Africa, our study contributes two near-complete genomes that provide additional evidence and strengthen the current understanding of the evolutionary history of this subgroup. Recent studies have revealed increased diversity within the LSDV cluster 1.2 [55]. Furthermore, several strains within the 1.2 cluster, such as the Pendik Turkey strain in 2014/2015 [8] and the Evros strain in Greece and Turkey [56], have been attributed to various outbreaks across Eurasia and South Asia. Moreover, historical analysis suggests that cluster 1.1 strains have been displaced by cluster 1.2 strains in South Africa, with an estimated substitution rate of 7.4 × 10^−6^ substitutions/site/year [57]. The rapid pace of LSDV mutations supports the diversity of sub-clusters within the 1.2 cluster, and this could mean changes in the virulence of the pathogen, transmission dynamics, or immune response in affected cattle populations. This has great implications for vaccine effectiveness and the ability of several regions to curb outbreak cases. For this reason, we suggest the need for polyvalent vaccines or regionally based vaccines coupled with effective control of transboundary cattle movement. Our findings also highlight the value of whole-genome sequencing in uncovering regional diversity and the need to revisit current classification strategies, which put underrepresented African strains into consideration. By doing so, better genomic and genetic information can be gathered to support the development of effective vaccines.

The findings from our study revealed a low number of substitutions and indels in the Cameroonian and Nigerian LSDV genomes compared with other representative strains from the 1.2 cluster (Dagestan2015, Warmbaths2000, and Israel2012), suggesting genomic conservation and evolutionary stability within this regional sub-lineage. Conversely, marked divergence from strains in Europe, Asia, and Southern Africa reflects the continuous evolution of the strains in these regions. For example, multiple strains from South Africa belong to clusters 1.1 and 1.2 [58], and LSDV strains from Russia, India, and China have representatives across multiple LSDV clusters [55,59,60]. This could explain the relatively low report of mortality and morbidity associated with LSDV in the western and central African region [61] due to better adaptation among host animals as opposed to the recurrent outbreaks within Eurasia and Asia [2,62], where recent outbreaks, especially in Southeast Asia, have shown unusually high mortality rates [63]. However, given that this study was a one-off, with limited full genomes assembled, there is a need for continued surveillance in Western and Central Africa to detect the true epidemiological picture of the disease in this region, as well as the need for broader African genomic datasets to better define LSDV’s global diversity and inform phylogeographic analyses.

The identification of non-synonymous mutations in several LSDV genes, particularly those involved in immune modulation and antigen presentation, carries important implications for vaccine design. Key mutated genes identified in this study, including LSDV012, LSDV144, LSDV145, and LSDV151, encode ankyrin repeats and Kelch-like proteins. These proteins are known to suppress host immune factors, such as interferon-induced proteins like IFIT1, facilitating immune evasion [41]. Similar mutations have been observed in other LSDV strains. Unique Kenyan-like LSDV strains circulating in India exhibited truncated versions of Kelch-like proteins encoded by LSD_019 and LSD_144, which may influence virulence and host range [64]. Supporting this, a functional study in sheeppox virus demonstrated that deletion of the Kelch-like gene SPPV-019, a homolog of LSDV019, led to marked in vivo attenuation, with infected sheep exhibiting dramatically reduced clinical symptoms, viremia, and virus shedding, showing the role of Kelch-like genes in regulating capripoxvirus virulence and immune modulation [65]. This could imply that strains carrying this truncation or deletion in the Kelch protein gene are relatively less virulent. Moreover, the role of Kelch protein deletion in reducing the virulence of the virus makes it important in vaccine design. A recent study demonstrated that vaccinia virus strains lacking the C2 Kelch-like gene not only exhibited attenuation in vivo but also induced a stronger CD8^+^ T cell memory response and improved protection against viral challenge, compared to control strains retaining the gene [66]. These findings highlight the functional significance of Kelch-domain proteins in immune modulation and virulence, and their deletion offers a promising strategy for the design of live attenuated vaccines that are both safer and more immunogenic.

Additionally, the study demonstrated that the Cameroonian strains do not fall into established sub-lineages, indicating genomic divergence from strains used for the development of vaccines such as the Neethling strain [67]. If the immunodominant regions of the virus, especially within the envelope proteins and core virion proteins, have accumulated substantial mutations, current vaccines may offer reduced protection. For instance, LSDV027, a gene encoding the extracellular enveloped virus (EEV) maturation protein, is key for virus dissemination and immune recognition [68]. Experimental data from the vaccinia virus provide further support for this concern. A study by Gurt et al. [69] found that the introduction of a single amino acid mutation in the B5R EEV envelope glycoprotein (WR.c3) induced lower titers of antibodies, suggesting that alterations in EEV surface proteins may impair humoral immune responses. This further supports the need for polyvalent vaccines or regionally based vaccines coupled with effective control of transboundary cattle movement.

Missense mutations identified in key virulence-associated genes (LSDV140 and LSDV146) suggest potential modulation of the virus’s pathogenic behaviour. The LSDV140 gene, a homologue of the N1R/p28 protein found in other poxviruses, is known to suppress host cell apoptosis, thereby promoting viral survival [43]. Mutations in this gene may either enhance or impair its anti-apoptotic function, potentially influencing the severity and duration of infection. Similarly, LSDV146, which encodes a phospholipase-D-like protein involved in viral dissemination, could influence the efficiency of intra-host spread [45,53]. Changes in this gene may affect how extensively the virus invades host tissues, thereby shaping the clinical presentation of the disease. Collectively, these mutations may make the strain more virulent. A strain that replicates more rapidly and suppresses immune detection may elicit a weaker immune response while also causing more severe disease, thereby complicating efforts to achieve effective immunisation and long-term protection. It should, however, be noted that while all these mutations could potentially affect the virulence of the virus or its immunogenic activities, functional studies are required to directly link these mutations to phenotypic changes. Most positive samples were skin scabs, with the lowest positivity seen in oral/nasal swabs. This adds to the report that skin scabs could be the most reliable sample for LSDV detection [7,70]. This implies that while it would be easy to collect oral/nasal swabs, their lower yield suggests they should be complemented with other sample types in outbreak investigations. Conversely, in a few cases, sequencing detected a small number of reads mapping to LSDV in samples that tested negative by qPCR. This discrepancy can occur because the region captured during sequencing may not overlap with the specific target region amplified by the qPCR primers. Furthermore, given that the reads under consideration in our study were very few relative to the large genome size (~151 kbp), such findings highlight that a handful of reads may be insufficient to infer meaningful viral presence and should be interpreted with caution.

This study had a few limitations. Firstly, although animals were purposively sampled by experienced veterinarians based on the presence of nodular skin lesions typical of LSD, similar clinical presentations can occur with other conditions, such as pseudocowpox [71] or dermatophilosis [72]. This diagnostic overlap may partly explain the low number of molecularly confirmed LSDV cases outside Cameroon, and highlights the urgent need for rapid, reliable field-based diagnostic tools. Furthermore, the Nigerian sampling was restricted to a single southwestern state and focused on abattoirs, which may not accurately represent the wider epidemiology in the country. These limitations should be considered when interpreting our findings, and they highlight the importance of expanding genomic surveillance across multiple sites and improving diagnostic capacity for LSDV in endemic regions. These limitations highlight the challenges of clinically distinguishing LSD from similar skin conditions and the restricted sampling approach used, particularly in Nigeria.

LSDV was also detected in flesh flies (*Sarcophaga* spp.). Although various arthropods have been linked to LSDV transmission, this is the first report of LSDV in flesh flies. Due to their feeding on exposed wounds [73], it suggests that other arthropods might also mechanically transmit LSD. Therefore, comprehensive vector control strategies are essential to prevent LSDV spread, especially in regions where arthropods are primary vectors, during disease outbreaks.

## 5. Conclusions

In conclusion, our study identified further divergence within the 1.2 cluster with the possibility of a western/central African sub-lineage. Moreover, the presence of high-impact mutations in genes associated with viral replication and immunogenicity may influence how these Cameroonian strains interact with the host immune system and could have implications for vaccine performance, although experimental studies are needed to confirm these effects. Our study also identified flesh flies as a novel arthropod involved in LSDV transmission. We, therefore, recommend the development of polyvalent vaccines that take into consideration the peculiarities in the antigenicity of the different LSDV strains circulating in the different regions of the world where LSDV outbreaks have been recorded. It is important to note that sampling in Nigeria was done in one southwestern state and in abattoirs alone. Therefore, the result from this study might not reflect the true picture of the epidemiology of LSDV in Nigeria.

## Figures and Tables

**Table 1:** Distribution of sample types collected from suspected cases of LSD in cattle across Benin, Cameroon, and Nigeria.

Country	Nodule Aspirate (*n*, %)	Skin Scab (*n*, %)	Oral & Nasal Swab (*n*, %)	Total (*n*, %)
Benin	0 (0)	40 (48.2)	43 (51.8)	83 (27.9)
Cameroon	10 (10.6)	28 (29.8)	56 (59.6)	94 (31.6)
Nigeria	0 (0)	60 (50.0)	60 (50)	120 (40.4)
Total	10 (3.4)	128 (43.1)	159 (53.5)	297 (100.0)

**Table 2:** Detection of LSDV DNA in pools of different fly species collected from sampled sites.

Country	Type of Fly	Number of Pools	LSDV PCR Positive Pool	Illustration
Cameroon	Green Bottle Fly	2	0	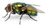
	House Fly	1	0	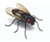
	Blue Bottle Fly	1	0	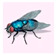
	Flesh Fly	1	1	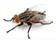
Nigeria	House Fly	2	0	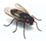
	Green Bottle Fly	1	0	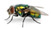
	Black Scavenger Fly	1	0	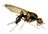
	Tachinid Fly	1	0	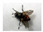
Benin	House Fly	8	0	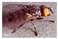
	Picture Wing Fly	3	0	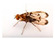
	Flesh Fly	4	0	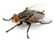
	Green Bottle Fly	4	0	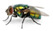
	Picture Wing Fly	2	0	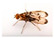
	Tachinid Fly	2	0	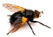
	Sweat Fly	1	0	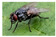
	Black Scavenger Fly	1	0	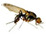
	Syrphid Fly	2	0	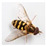
	Horn Fly	3	0	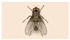
	Blue Bottle Fly	1	0	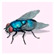

**Table 3:** Association between country, sample type, and LSDV positivity among clinically suspected cattle cases.

Category	Variable	Total	PCR Positive	%Positivity	χ2	*p* Value
Country (*n* = 172 cattle)	Benin Rep	55	1	1.8	19.01	<0.001
	Cameroon	57	12	21.1		
	Nigeria	60	1	1.7		
Sample type (*n* = 297)	Nodule aspirate	10	1	10	3.9	0.139
	Skin scab	128	12	9.4		
	Oral & Nasal Swab	159	6	3.8		

**Table 4:** Summary of sequence variations across LSDV genomes as compared with the reference genome (NC003027).

Strain	Single Base Substitutions or SNPs	Insertions	Deletions
AF409137.1_Warmbaths2000	123	78	24
CAYDCAT29	124	14	22
CAYDCAT30	136	24	25
KX894508.1_Israel2012	134	39	45
MH893760.2_Dagestan2015	147	20	15
OK318001.1_Nigeria2018	134	44	25

**Table 5:** Functional classification of genetic variants in Cameroonian LSDV genomes.

Metric/Variant Type	CAYDCAT29	CAYDCAT30
Missense variant	40	43
Stop gained	0	1
Synonymous variant	86	85
Upstream gene variant	3	4
Number of genes with variations	82	81
Unique genes with variations	1 (LSDV154)	0
Genes with ≥2 variations	23	25
Genes with non-synonymous variations	35	37

## Data Availability

Sequences derived from this study have been submitted to GenBank under the accession numbers PV963838 and PV963839 with Bioproject PRJNA1290111.

## Supplementary Materials

The following supporting information can be downloaded at https://www.mdpi.com/article/10.3390/pathogens14090922/s1, Table S1: qPCR result; Table S2: LSDV read counts from sequenced samples; Table S3: coverage and mean read depth report; Table S4: CAYDCAT29 annotated mutations; Table S5: CAYDCAT30 annotated mutations.

## Abbreviations

The following abbreviations are used in this manuscript:

Abbreviation	Full Meaning
LSDV	Lumpy Skin Disease Virus
DNA	Deoxyribonucleic Acid
RNA	Ribonucleic Acid
qPCR	Quantitative Polymerase Chain Reaction
qRT-PCR	Quantitative Reverse Transcriptase PCR
CT	Cycle Threshold
EEV	Extracellular Enveloped Virus
ORF	Open Reading Frame
SNP	Single-Nucleotide Polymorphism
Indel	Insertion or Deletion Mutation
NCBI	National Center for Biotechnology Information
MAFFT	Multiple Alignment using Fast Fourier Transform
IQTREE2	Efficient software for phylogenomic inference
iTOL	Interactive Tree of Life
BWA	Burrows-Wheeler Aligner
NUCmer	Nucleotide MUMmer (alignment software)
SnpEff	SNP Effect Predictor
PCR	Polymerase Chain Reaction
SPSS	Statistical Package for the Social Sciences
MUMmer	Maximal Unique Match aligner
AWK	A pattern scanning and processing language
VirCapSeq-VERT	Virus Capture Sequencing for Vertebrates
IGH	Institute of Genomics and Global Health
USA	United State of America
